# Early Diagnosis of Lung Cancer in the United Arab Emirates: Challenges and Strategic Recommendations

**DOI:** 10.3390/clinpract11030082

**Published:** 2021-09-15

**Authors:** Humaid O. Al-Shamsi, Hassan Jaffar, Bassam Mahboub, Faraz Khan, Usama Albastaki, Sayed Hammad, Ashraf Al Zaabi

**Affiliations:** 1 Department of Oncology, Burjeel Cancer Institute, Burjeel Medical City, Abu Dhabi 111499, United Arab Emirates; 2 Innovation and Research Center, Burjeel Cancer Institute, Burjeel Medical City, Abu Dhabi 111499, United Arab Emirates; 3 College of Medicine, University of Sharjah, Sharjah 999041, United Arab Emirates; 4 Emirates Oncology Society, Dubai 22107, United Arab Emirates; 5 Sheikh Khalifa Specialty Hospital, Ras Alkhaima 999041, United Arab Emirates; hjaaafar@hotmail.com; 6 Rashid Hospital, Dubai 111499, United Arab Emirates; drbassam_mahboub@yahoo.com (B.M.); usamauae@gmail.com (U.A.); 7 American Hospital, Dubai 111499, United Arab Emirates; fkhan@ahdubai.com; 8 Dubai Hospital, Dubai 111499, United Arab Emirates; stirmazy@icloud.com; 9 Zayed Military Hospital, Abu Dhabi 111499, United Arab Emirates; ashrafalzaabi@hotmail.com

**Keywords:** lung cancer, United Arab Emirates, screening, referral pathway

## Abstract

In the United Arab Emirates (UAE), lung cancer (LC) was the third leading cause of deaths due to cancer in 2017. Around 80% of the patients in the UAE are diagnosed at a late stage, rendering the treatment less effective in improving survival outcomes. Lack of awareness of disease symptomatology, deficient screening initiatives, misdiagnosis, and delayed referral to the specialist are contributing factors for delayed diagnosis. Effective screening at a primary care setting can be crucial for early diagnosis, referral to specialists, and enhancing patient outcomes. It is important to establish screening and referral guidelines through which each suspected case can be identified and provided timely intervention. Although the international screening and referral pathway framework are comprehensive, several regional barriers need to be addressed before they can be adapted at the national level. A group of LC experts from the UAE deliberated on issues like delayed diagnosis of LC and strategic recommendations for overcoming the challenges. The discussion was based on the review of the published evidence, international and regional guidelines for screening and early diagnosis of LC. Herein, we present a guideline, endorsed by the esteemed panel of experts, for aiding early diagnosis and optimizing the management of LC in the UAE.

## 1. Introduction

Lung cancer (LC) continues to be the single leading cause of cancer deaths worldwide [1]. It was the second most common cancer with 2.21 million cases and 1.8 million deaths in 2020 [2]. Non-small-cell lung cancer (NSCLC) accounts for about 85% cases of primary LC, with adenocarcinoma as one of the most common histologic subtypes [3]. About 80% of NSCLC is diagnosed at an advanced stage, with the 5-year survival rate being 0% to 10% for patients with stage IVA-IVB [4,5]. In the United Arab Emirates (UAE) in the year 2017, LC had an incidence of 7.76% (33/425) in males and 1.02% (7/680) in females. LC was the third common cause of cancer mortality in both males (10.8%) and females (5.5%), with an estimated average of 80 deaths (8.4%) in 2017 [6]. In 2018, the estimated incidence of LC in UAE was 4.0%, with mortality 8.8% and the age-standardized incidence rate 6.3% [1,7,8].

The major risk factor associated with all histological types of LC is tobacco smoking [9]. There is a 20- to 50-fold greater risk of developing LC in continuous smokers compared to never-smokers, with duration of smoking being the strongest determinant of LC risk. Other tobacco products such as cigars, cigarillos, pipes, bidi, hookah and water pipes (shisha smoking), and involuntary smoking (workplace exposure, household exposure, childhood smoking exposure) are also associated with a higher risk of developing LC. The excess risk is in the order of 20% to 30% for a nonsmoker married to a smoker. Risk factors such as genetic susceptibility, advanced age (55 to 74 years), poor diet, indoor and outdoor air pollution, occupational exposure to carcinogens (such as asbestos, silica, radon, heavy metals, polycyclic aromatic hydrocarbons, ionizing radiation), and chronic lung inflammation may act independently or in association with tobacco smoking to increase the risk of LC [9,10].

A large population-based study has reported that family history of LC is a risk factor for developing LC (hazard ratio (HR): 1.95, 95% confidence interval (CI), 1.31 to 2.88) [11]. According to this study, the association was considerably stronger in women (HR: 2.65; 95% CI, 1.40 to 5.01) compared to men (HR: 1.69, 95% CI, 1.03 to 2.78), and in nonsmokers than in smokers (HR: 2.48, 95% CI, 1.27 to 4.84 and HR: 1.73; 95% CI, 0.99 to 3.00). Molecular studies have demonstrated epidermal growth factor receptor (EGFR) mutation as an oncogenic driver mutation of LC. According to the molecular epidemiological studies, the EGFR mutation frequency detected in Gulf patients is higher (28.7%) than that shown in white populations (15%), but still lower than the frequency reported in Asian populations [12].

In the Middle East and North Africa (MENA) region, approximately 46% of the population smoke and the number continues to increase. Although most Arab countries have adopted the Framework Convention on Tobacco Control, widespread use of cigarettes and water pipes persists in the MENA region. Current data show that the smoking prevalence in the UAE is 28.0% in males and 0.9% in females. The use of tobacco as well as the incidence of LC has been steadily increasing in the MENA region, including the UAE [1].

An epidemiological study by Jaafar et al. revealed that, of the 962 cases of LC from 4 Gulf countries, more than half of these NSCLC cases were observed in the age group of 45 to 65 years, and the majority (76.3%) were diagnosed in the advanced stage (mostly Stage IV) [13]. In the Gulf nations (including the UAE), delayed presentation and diagnosis, lack of an early screening and referral program may contribute to delayed diagnosis of LC.

The uncontrolled tobacco epidemic, delayed diagnosis, and the increasing incidence of LC point toward an urgent need for a screening and referral program for the early detection. An expert panel steering committee meeting was therefore convened to identify the current gaps and challenges in early detection of LC in the UAE.

## 2. Methodology

A panel of experts from pulmonary medicine and allied fields discussed the current practice, and limitations in the screening of LC in the UAE. All experts interacted in contextual question-based discussions and proposed relevant literature to be appraised during the meeting. A systematic approach toward key issues was taken including: (1) a delay in diagnosis of LC in the UAE, (2) limited data on incidence and prevalence of LC, and (3) effective screening initiatives and recommended referral pathway. The available information, together with expert opinions, was compiled to draw a consensus. 

For the review of evidence and development of recommendations, a literature search was conducted using MEDLINE and EMBASE databases. Several keywords including “lung cancer,” “diagnosis,” “screening,” “referral pathway,” “DOH,” “PLCOm2012,” “screening program,” “survival,” and “United Arab Emirates,” were used for in-depth literature search on the topic. The results presented in the literature were reviewed, and the recommendations were developed.

## 3. Screening, Diagnosis, and Referral Practices for Lung Cancer: Current Scenario in the UAE

LC is known to be associated with poor patient survival if diagnosed in advanced stages (Stage III and IV) [14]. It is important to detect LC at an early stage to offer effective treatment options, including surgical resection, which may greatly improve survival outcome [14]. Early diagnosis followed by surgical resection offers a favorable prognosis with a 5-year survival rate of 70% to 90% for small, localized tumors (Stage I). However, only about approximately 25% of patients are detected at Stage I and Stage II [14].

Screening high-risk individuals with low-dose computed tomography (LDCT) results in significant reduction in mortality and helps in the diagnosis of a larger proportion of patients with LC at earlier stages [15]. However, compliance with screening is believed to be quite low throughout the UAE [10]. There is a lack of general awareness about the link between smoking and LC [1]. LC screening is implemented mostly in Abu Dhabi; it is, however, not yet implemented in the rest of the UAE [16]. Limited regional data makes it difficult to estimate the prevalence of early-stage LC in the UAE [1].

While individual physicians may screen high-risk patients, the LC screening guidelines are not followed uniformly by all physicians. In addition, screening with LDCT is not thought to be risk-free because of the possibility of unnecessary procedures, follow-ups, and anxiety for the patients. As screening with LDCT often reveals benign, noncancerous findings, the yield and cost-effectiveness of the procedure has not been well studied in the region. Overlapping symptoms with other respiratory disorders and a differential diagnosis of pneumonia or tuberculosis can further delay the diagnosis of LC [17]. Moreover, misinterpretation of imaging reports and failure to diagnose LC by primary care physicians (PCPs) leads to delayed referral to specialists and missed opportunities for optimal management [1]. 

Lack of homogenous or standard referral pathway for all the hospitals is a critical challenge. There exists a lack of awareness and inadequate training of PCPs on disease symptomatology and progression. Early symptoms of LC can also be missed in the case of patients with comorbidities [17]. In the UAE, the referral of a patient of LC from a chest physician to the oncologist is often unhindered. However, the delay happens at the diagnosis stage, i.e., from a general practitioner (GP)/PCP to the pulmonologist [18]. Thus, inadequate communication between PCPs and LC specialists, inaccessibility to specialists, and lack of guidance regarding the referral pathway severely hamper the outcomes of patients with LC.

## 4. Guidelines for Early Detection of Lung Cancer

From 2002 to 2004, a landmark randomized controlled trial, the National Lung Screening Trial (NLST), enrolled 53,454 current or former heavy smokers with a smoking history of at least 30 pack years and without signs, symptoms, or history of LC. This trial compared the efficacy of two imaging techniques (LDCT and chest radiography) to determine whether screening with LDCT could reduce mortality from LC. The study findings revealed that there were 247 deaths from LC per 100,000 person-years in the LDCT group and 309 deaths per 100,000 person-years in the radiography group. This represented a 20% (95% CI, 6.8 to 26.7; *p* = 0.004) reduction in mortality from LC with LDCT screening [15].

Following the NLST, another randomized controlled trial, the Nederlands–Leuvens Longkanker Screenings Onderzoek (NELSON), randomized 15,792 adults with 50 to 75 years of age, current or former smokers, with smoking history of >15 cigarettes daily for >25 years or >10 cigarettes daily for >30 years and ≤10 years after quitting, to four rounds of LDCT at baseline and 1-, 2- and 2.5-year intervals versus no screening. The results revealed that 58.6% of the screening-detected cases were in the early stages (IA and IB). At the 10-year follow-up, the reduction in LC mortality was 24% and 33% among men and a small sample of women, respectively [19].

Based on the findings of the NLST and the NELSON trial, the American Cancer Society and the National Comprehensive Cancer Network (NCCN) (United States) as well as the National Health Service (United Kingdom) released LC screening guidelines targeting current or past smokers aged 55 to 74 years with a smoking history of 30 pack years [20,21,22,23]. As per the Cancer Research United Kingdom, >80% of patients with LC will survive for at least a year if diagnosed at the earliest stage compared to ~15% for people diagnosed at the advanced stage of the disease [14]. Several other countries such as Canada and Australia have also issued guidelines to GPs for referral of patients at high risk of developing LC for imaging tests (chest radiograph and LDCT) [24,25]. 

In March 2021, the United States Preventive Services Task Force published substantial revisions in LC screening recommendations. Based on these revised guidelines, annual screening for LC with LDCT is recommended in adults aged 50 to 80 years who have a 20-pack-year smoking history and currently smoke or have quit within the past 15 years [26].

The NLST criteria have been widely used to identify persons eligible for screening of LC. In addition, the risk prediction model, PLCOm2012, (developed by Tammemägi et al. in 2013) was found to be highly accurate in predicting the 6-year risk of LC. This risk prediction model considers several factors such as age, race, education, body mass index, chronic obstructive pulmonary disease (COPD), personal history of cancer, family history of LC, smoking status, and duration since cessation of smoking [27]. The risk of LC increases with age, black race, lower socioeconomic status, lower body mass index, history of COPD, personal history of cancer, family history of LC, current smoking, increased smoking intensity and duration, and shorter time since quitting in former smokers [27]. This model helps stratification of patients being screened for LC into high- and low-risk strata and guides decision making regarding screening interval. It may also help to reduce the number of screenings, improve cost-effectiveness, and decrease the radiation exposure [27,28]. A PLCOm2012 6-year risk score of ≥1.51% is the threshold for eligibility for the screening intervention. LDCT should be performed in patients with a score of ≥1.51% [28]. A biennial screening program for individuals aged 55 to 74 years with a minimum 6-year LC risk 1.51% (using the PLCOm2012) has been reported to be the most cost-effective form of screening program [27,28,29]. The UAE government aims to reduce mortality related to LC, as it is the leading cause of death in the UAE. Hence, the Department of Health (DOH)–Abu Dhabi formulated specifications for a LC screening program (2018) for the Emirate of Abu Dhabi, highlighting the minimum service specifications to ensure that high-risk candidates screened for LC receive quality and safe care and timely referral for diagnosis and/or treatment [16].

In Abu Dhabi, the LC screening program aims for a participation rate (percentage of subjects who have a screening LDCT as a proportion of the population at risk) of >10% and retention rate (percentage of LDCT-negative screened subjects who follow up screening) >50%. There are no official data about the uptake of the screening in Abu Dhabi. 

## 5. Recommendations for Screening and Referral for Lung Cancer in the UAE

All the experts deliberated the need to establish clear guidelines through which every suspected case of LC should be identified, referred to a specialist for early diagnosis, with subsequent management based on the recommendations.

Compliance to guidelines for screening and early referral to a specialist for diagnosis and timely treatment are known to greatly improve the patient outcome. Several observational studies have shown that LDCT of the lung detects more nodules and LC, including early-stage cancers, than chest radiography.

PCPs and GPs are often the first to come across patients with a history of smoking and exposure to other risk factors of LC. Therefore, based on the criteria followed by the NLST, several organizations such as the American Cancer Society, American College of Chest Physicians, American Society of Clinical Oncology, NCCN, and The Royal Australian College of General Practitioners have recommended a pathway to help GPs issue early screening imaging referrals (chest X-ray and chest LDCT). A proposed screening guideline for the UAE has been adopted from these guidelines [20,22,25]. According to these guidelines, people aged between 55 and 74 who are current or former heavy smokers with at least one cigarette pack daily for 30 years or two packs for 15 years, exposure to certain pollutants, hazardous substances like asbestos, diesel exhaust, previous lung disease, and a family history of LC are at increased risk for LC. These patients should be screened for LC with annual chest scans or LDCT. A PLCOm2012 6-year risk score of ≥1.51% is the threshold for biennial screening by LDCT [29].

For patients with specific symptoms suggestive of LC, like hemoptysis, guidelines suggest that GPs/PCPs must issue an urgent referral for imaging (chest X-ray and chest LDCT) as well as a referral to a specialist linked to a LC multidisciplinary team (MDT) with concurrent computed tomography (CT). The referrals follow an algorithmic approach and are tailored to suit each individual case.

Physicians must also keep in mind that LC can present in younger individuals and in the absence of risk factors [21]. It is of the utmost importance to develop GP referral guidelines focusing on risk factors, urgent chest X-ray indications, and patients’ criteria for referral (Table 1).

**Figure 1:**
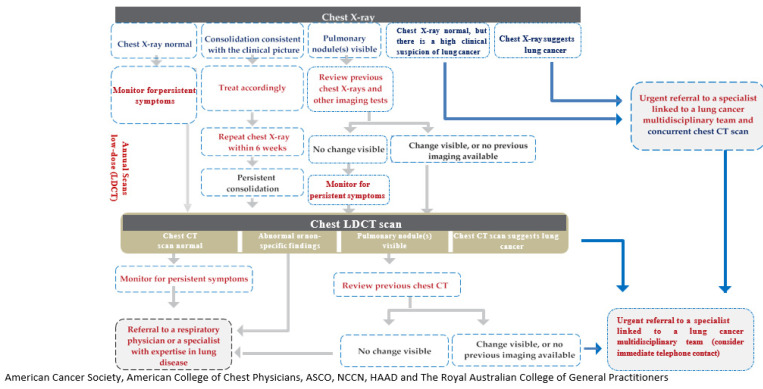
Recommended referral pathway for patient with suspected lung cancer. CT: computed tomography; LDCT: low-dose computed tomography.

The experts also focused on prompt referral of symptomatic patients to diagnostic and treatment services. It is recommended to perform urgent chest X-ray in the presence of specific symptoms suggestive of LC. If chest X-ray is normal, the patient must be monitored for symptom resolution and must be referred for LDCT if symptoms persist. In the event of persistent consolidation or visible pulmonary nodules (which show changes when compared to previous X-rays), the patient must be referred for LDCT. If the LDCT is normal, or has abnormal nonspecific findings, or has visible pulmonary nodules with no changes when compared to previous CTs, the patient must be referred to a respiratory physician. However, if pulmonary nodules show changes when compared to previous CTs, urgent referral must be done to a specialist linked to a LC MDT. Key performance indicators measuring the time from the abnormal radiological findings to the treatment initiation can be implemented to monitor the effectiveness of the referral system (Figure 1).

## 6. Conclusions

It is indeed ironical that the principal cause of one of the most lethal cancers is preventable in most cases. Awareness about tobacco smoking being the single, most common causative factor for developing LC needs to be propagated among the people as well as healthcare professionals. Several screening programs for LC have reported improved survival outcomes. These programs, especially targeting high-risk patient populations, can be effective tools for early diagnosis, educating the primary healthcare professionals as well as creating awareness among the masses. They can also help in generating population-based epidemiological data. Along with screening, the PCPs and the pulmonologists need to be armed with a referral pathway for patients with suspected LC. Consultation with an MDT could be the key to providing timely and accurate diagnosis and treatment. There have been ground-breaking advances in the management of LC in the last few years. The proposed national level screening program and the recommended referral pathway are steps in the right direction for setting the stage for the patients to benefit from these therapeutic advances and receive optimum LC care in the UAE.

## Figures and Tables

**Table 1:** Guideline for Screening and Diagnostic Approach [10,20,22,25,29,30,31,32].

Risk Factors for Lung Cancer	Indications for Urgent Chest X-ray	Indication for LDCT
**Personal factors:** Age: between 55 and 74 Family history of lung cancer (LC)	Offer an urgent chest X-ray (to be performed within 2 weeks) to assess for LC in people aged 40 and over if they have 2 or more of the following unexplained symptoms, or if they have ever smoked and have 1 or more of thefollowing unexplained symptoms: Cough Fatigue Shortness of breath Chest pain Weight loss Appetite loss	People aged between 55 and 74 who are current or former heavy smokers with at least one cigarette pack daily for 30 years or two packs for 15 years have been recommended for annual scans with low-dose computed tomography (LDCT) A PLCOm2012 6-year risk score of ≥1.51%
**Lifestyle factors:** Tobacco smoking: Current or former heavy smokers: at least one cigarette pack daily for 30 years or two packs for 15 years		A patient whose chest X-ray is suspicious of LC including: A nodule or mass Multiple pulmonary nodules Nonresolving pleural effusion Mediastinal or contralateral hilar adenopathy Interstitial infiltrates Slowly or nonresolving pneumonia or consolidation Fibroapical disease suggesting possible tuberculosis Unexplained elevated diaphragm
**Environmental factors:** Passive smoking Radon exposure Occupational exposure, e.g., asbestos, diesel exhaust Air pollution	Consider an urgent chest X-ray (to be performed within 2 weeks) to assess for LC in people aged 40 and over with any of the following: Persistent or recurrent chest infection Finger clubbing Supraclavicular lymphadenopathy or persistent cervical lymphadenopathy Chest signs consistent with LC Thrombocytosis	A patient who has hemoptysis, or other symptoms, which are concerning or persistent, even if their chest X-ray is normal. Are aged 40 and over with unexplained hemoptysis.

## Abbreviations

CI: Confidence interval; CT: Computed tomography; DOH: Department of health; EGFR: Epidermal growth factor receptor; GP: General practitioner; HR: Hazard ratio; LC: Lung cancer; LDCT: Low-dose computed tomography; MDT: Multidisciplinary team; MENA: Middle East and North Africa; NSCLC: Non-small-cell lung cancer; NCCN: National comprehensive cancer network; NLST: National lung screening trial; NELSON: Nederlands–Leuvens Longkanker Screenings Onderzoek; PCP: Primary care physician; UAE: United Arab Emirates; USPSTF: United States preventive services task force
